# A Mixed Methods Exploration of Young Women’s Agency and Mental Health during COVID-19 in Low-Income Communities in Mumbai, India

**DOI:** 10.3390/ijerph21081007

**Published:** 2024-07-31

**Authors:** Marie A. Brault, Melissa F. Peskin, Anastasia N. Jones, Amrita Saikia, Rinchen O. Bhutia, Sai Sammitha Cheruvu, Vaishali M. Jagtap, Rajendra Singh, Poornima Nair, Rajesh Vedanthan, Sten H. Vermund, Shubhada Maitra

**Affiliations:** 1 Department of Health Promotion and Behavioral Sciences, The University of Texas Health Science Center at Houston School of Public Health, San Antonio, TX 78229, USA; 2 Department of Health Promotion and Behavioral Sciences, The University of Texas Health Science Center at Houston School of Public Health, Houston, TX 77030, USA; melissa.f.peskin@uth.tmc.edu; 3 Department of Epidemiology, Human Genetics and Environmental Health, The University of Texas Health Science Center at Houston School of Public Health, San Antonio, TX 78229, USA; anastasianjones@gmail.com; 4 Jamsetji Tata School of Disaster Studies, Tata Institute of Social Sciences, Mumbai 400088, India; amrita.saikia@gmail.com; 5 Centre for Public Health, School of Health Systems Studies, Tata Institute of Social Sciences, Mumbai 400088, India; mm2018ph007@tiss.edu; 6 Department of Epidemiology, Human Genetics and Environmental Health, The University of Texas Health Science Center at Houston School of Public Health, Houston, TX 77030, USA; sammithacheruvu@gmail.com; 7 Independent Researcher, Mumbai 400074, India; vaishali_465@yahoo.com; 8 International Center for Research on Women (ICRW) Asia Office, New Delhi 110018, India; rajendrasingh.research@gmail.com; 9 Health and Disability, Apnalaya, Mumbai 400071, India; poornima.nair@apnalaya.org; 10 Department of Population Health, New York University Grossman School of Medicine, New York, NY 10016, USA; rajesh.vedanthan@nyulangone.org; 11 Department of Epidemiology of Microbial Diseases, Yale School of Public Health, New Haven, CT 06510, USA; sten.vermund@yale.edu; 12 Centre for Health and Mental Health, School of Social Work, Tata Institute of Social Sciences, Mumbai 400088, India; shubhada@tiss.edu

**Keywords:** adolescent girls and young women, COVID-19, agency, mental health, financial insecurity, health emergency, India

## Abstract

Introduction: Adolescent girls and young women (AGYW) in India face additional health inequities compared to their male peers, as gender norms constrain agency for prevention and self-care. The onset of the COVID-19 pandemic and associated lockdowns deepened health inequities and often worsened mental health, but the impacts on agency are unclear. This exploratory sequential mixed methods paper examined mental health and COVID-19 elements that exacerbated or mitigated adverse consequences for AGYW in low-income communities in Mumbai. Methods: We conducted semi-structured interviews with AGYW (aged 15–25 years; N = 60) and adults (parents, healthcare providers, community-based organization representative; N = 30). We administered a structured survey to AGYW (N = 150) to assess health concerns, depression and anxiety symptoms (using the PHQ-8 and GAD-7 scales), and experiences during COVID-19. We analyzed qualitative data using the constant comparative approach in Atlas.ti, and quantitative data using R and SPSS. Results: Qualitative data revealed that AGYW faced stressors and had limited agency during lockdowns due to limited access to education, financial insecurity, and community violence. Quantitative data indicated that limited agency in the context of COVID-19 was significantly associated with depression and anxiety. Financial resources to address COVID-19 created new employment and leadership opportunities for AGYW to become COVID educators and preschool teachers; participation in these opportunities was associated with less anxiety. Discussion: Pandemic stress was difficult for low-income AGYW in Mumbai. Mitigating programs for COVID-19 control helped address acute needs and enable capabilities. Exploring similar themes among a broader population of youth can help design strategies and opportunities for young people in low-income communities during health emergencies.

## 1. Introduction

Adolescent girls and young women’s (AGYW) health inequities may be due to limited agency, or limited ability to conceptualize and actualize goals, related to education, healthcare, and employment [1,2,3,4,5,6]. Inequitable gender norms that cause women and girls to be treated differently from male peers in social and interpersonal relations are linked with AGYW higher rates of anxiety, depression, and suicidality, compared to young men [7,8]. The impacts of COVID-19 on AGYW lives have been widely, but variably reported in the literature. Researchers have consistently found that COVID-19 lockdowns and financial hardship worsened mental health for youth in India and AGYW in particular [9,10,11,12,13,14,15,16]. The relationships between educational restrictions in COVID-19 and mental health are also more consistent, suggesting that educational disruptions produced disproportionate stress and educational disruption for AGYW [15,16,17,18,19]. However, the literature on COVID impacts on gender equity and female agency in low- and middle-income countries (LMICs) is less consistent. In some settings, girls and women experienced increased financial insecurity and less ability to rejoin the workforce [20,21]. Other studies reported some level of resilience due to participation in financial empowerment programming, such as microcredit and women-led self-help groups [20,21,22]. AGYW’s experiences of gender-based violence and harassment, i.e., verbal, physical, or emotional abuse due to gender, in the context of COVID-19 have been described variably. Some studies report greater victimization, and others find little change compared to the pre-COVID-19 period [23,24,25,26,27]. As countries seek to address COVID impacts on AGYW, and plan for ongoing and future health and humanitarian crises, clarifying the relationships between female agency and physical and mental well-being can guide the development of policies and programming [11,21,28].

Global trends in gender inequity, female agency, and poor female physical and mental health are mirrored in India. Despite the existence of policies and programs promoting gender equity, AGYW experience disproportionate rates of gender-based violence, less access to education and healthcare, less choice concerning the timing of marriage, and neglect of personal aspirations [29,30,31,32,33,34,35,36]. These experiences, in turn, are adversely associated with mental health [2,29,35,37,38,39,40,41,42]. However, it is unclear how COVID-19 may have impacted AGYW’s agency or mental health, and the implications of this for youth-centered health programming. India’s trajectory during COVID-19 was unique, given the size and diversity of the country, but may offer important lessons to other complex settings [43]. This sub-study sought to explore and elaborate on the relationships between COVID-19, female agency, and mental health in the context of a larger study to systematically adapt and pilot a peer support program for AGYW in low-income communities in Mumbai. We used a systematic adaptation framework that calls for an initial needs assessment to understand the context and determine components requiring adaptation [44]. Findings from this study not only inform ongoing efforts to implement youth-friendly sexual, reproductive, and mental health programming in India but may also inform similar efforts post-COVID-19.

## 2. Methods

In the needs assessment phase of a study to adapt and pilot programs to improve AGYW sexual and reproductive health (SRH) in low-income communities in Mumbai India, we employed an exploratory sequential mixed methods design. Qualitative data collection with AGYW and key adults in the communities provided insights into AGYW’s needs and assets in the context of COVID-19, and informed the measure selection in the subsequent quantitative survey, as well as the wording/translation of questions [45]. Quantitative data enabled us to test hypotheses concerning the relationship between agency, mental health, and COVID-19 that emerged from the qualitative themes.

### 2.1. Setting

We recruited AGYW in several low-income (officially designated “slum”) communities in northeastern Mumbai, all located in a ward (administrative section) that ranks low in literacy and life expectancy rates and ranks high in out-of-school youth, infant mortality, and gender-based violence [46]. The population of the study area is >500,000, and >60% are under 25 years old. The per capita monthly income is INR 2570 (Indian rupees) (≈USD 30). Other features of the communities are described elsewhere [46,47]. India’s first mandatory COVID-19 lockdown was from 25 March to 31 May 2020. The lockdownwas announced with little notice, and triggered substantial financial hardship among the urban poor [48,49,50]. The Delta variant of COVID-19 that emerged in April 2021 triggered additional lockdowns. Schools and colleges in Mumbai were closed or online from 2020–2021, requiring students to access coursework through smartphones or laptops for ≈18 months; not all students had access to these electronic options.

### 2.2. Qualitative Data Collection and Analysis

Between December 2021 through April 2022, we conducted in-depth interviews with AGYW between the ages of 15–25 years old (N = 60), and ‘key informant’ interviews (KIIs; N = 30) with adults working in the study area who had deep knowledge of community norms and supports and challenges for youth. Key informants included representatives from non-governmental organizations (NGOs), parents of AGYW, community health workers, and healthcare providers.

We conducted semi-structured interviews using a discussion guide (Supplementary Materials) to explore AGYW’s perceptions of their communities, experiences during COVID-19, health needs and care-seeking, SRH and psychosocial concerns, and elicit input on future interventions. The KII guide (Supplementary Materials) focused on AGYW health-seeking and needs, community programming for youth, social and familial norms and attitudes that influence AGYW’s lives, and input on interventions. The development of both guides was informed by our team’s previous work in the study area, as well as theories of youth-centered programming, female agency and gender equity, and implementation science [31,51,52,53,54,55,56,57,58,59,60,61].

We recruited interview participants using purposive and snowball sampling from the authors’ long-standing community networks. With KIIs, we sought a mix of roles and experience in the study area. For AGYW interviews, we sampled to ensure representation of neighborhoods, married/unmarried, and religions (Muslims, Hindus, and Buddhists). Sampling and interviews were continued until content saturation was reached. Interviews (40–60 min), were conducted in Hindi, Marathi, or English (per participant), recorded, transcribed, and translated into English, when needed.

Our 5-person team included Indian and U.S. students and faculty with public health, anthropology, qualitative methods, and adolescent health expertise. We used the constant comparative approach to conduct review and coding of data in ATLAS.ti, version 24 online. Initially, analysts coded the same set of transcripts independently and met to discuss and compare coding approaches and resolve discrepancies. Once we reached consensus on coding, we divided the remaining transcripts, such that two analysts coded each one independently, with regular meetings to discuss progress and emergent themes. Two analysts coded 75% of the interviews, establishing, and refining the codebook through five drafts, and group coding. The remaining 25% of the interviews were coded by one analyst. We used a framework approach with codes based on key study and conceptual factors of interest, with additional codes added as novel topics arose [62,63]. Through in-depth review of code reports, we conducted thematic analyses on the factors that shaped AGYW’s experiences and health; qualitative analyses also helped inform the selection of domains assessed in the quantitative survey.

### 2.3. Quantitative Data and Analysis

We administered a 30–40-min structured survey to a random sample of 150 AGYW aged 15–25 years old; 25 participants were recruited from six sub-sections of the study area to ensure broad representation of socioeconomic status and religion. Participants were identified and recruited by a community-based organization that conducts health and social programming in the area and maintains lists of households.

The structured survey assessed four domains related to the following: (1) demographic information; (2) SRH and mental health concerns and treatment seeking; (3) perceived quality of and experiences with healthcare; and (4) COVID-19 work and agency (Table 1). Agency was conceptualized in terms of mobility, ability to access healthcare and education, personal say in timing of marriage, educational and employment attainment, and equitable treatment in household responsibility and rules [47,52,64]. We assessed mental health using the PHQ-8 for symptoms of depression and GAD-7 for symptoms of anxiety [65]. We also assessed the frequency of two cultural concepts of mental health: *tenshun*, a generalized psychological distress and *ghabrahat*, a form of nervousness or anxiety [66,67,68]. All measures were validated, and translated into Hindi, the language of survey administration. A trained research consultant administered surveys using RedCap Mobile between April–July 2022 [69,70]. Survey data were reviewed for completeness at the time of the survey, and then uploaded to the University of Texas Health Science Center at Houston (UTHealth Houston) RedCap server for storage and management.

We cleaned and re-coded data as needed, and calculated scales using R and SPSS. We applied descriptive statistics for continuous and categorical variables. Variables were normally distributed and did not require transformation. For the variables of interest, we applied bivariate correlations.

### 2.4. Ethical Considerations

Study procedures were approved by the UTHealth Houston Committee for the Protection of Human Subjects (HSC-SPH-21-0834), the Tata Institute of Social Sciences IRB (2019–2024), and the Indian Council of Medical Research/Health Ministry Screening Committee (2020-10093). All participants provided verbal informed consent, and for unmarried participants < 18 years old, verbal consent was obtained from a parent/guardian.

## 3. Results

We identified three themes from the qualitative data, with associated sub-themes: (1) individual and familial stressors during COVID-19 lockdowns, (2) community-wide insecurity during COVID-19 lockdowns, and (3) new opportunities and supports emerging from COVID-19 (Table 2). Quantitative data confirmed significant relationships between COVID-19 experiences, agency, and mental health.

Key socio-demographic characteristics and descriptive statistics of the mental health variables of interest for the AGYW quantitative sample are in Table 3; characteristics were similar for the quantitative and qualitative samples. The mean age of the AGYW participants was 18.0 years old (median 17). Most participants were unmarried.

### 3.1. Individual and Familial Stressors during COVID-19 Lockdowns

#### 3.1.1. Educational Obstacles

AGYW described numerous challenges continuing their education during COVID-19 lockdowns and after schools were re-opened (see Table 2 for all quotes). During lockdowns, AGYW had difficulty accessing their lessons online. Young women often had to share smartphones or tablets and internet data with siblings and/or parents, and cope with poor network connectivity, limiting their ability to view lessons and ask questions. Some AGYW found that their household responsibilities increased, as family members spent more time at home, further limiting their studies. Others described how changing government curricula and educational policies created obstacles to obtaining credentials for employment, even with completion of studies.

#### 3.1.2. Financial Hardships

COVID-19 lockdowns in India were particularly challenging for the urban poor, as many lost access to informal sector employment. Young women and KIIs described family arguments and even domestic violence due to lost wages and food insecurity. Some families were forced to borrow money to pay for rent and necessities. Others made the difficult decision to send some family members to stay with extended family in rural villages. Transit to isolated, rural areas could be especially difficult for AGYW who may have found their studies and aspirations further limited.

#### 3.1.3. Stress Due to Illness

Young women’s concerns were compounded by fears that they or their family members may contract SARS-CoV-2. AGYW who experienced COVID-19 infection, or had family members become ill, described the distress caused by the symptoms of the illness, as well as the strain of caring for family members. AGYW also feared passing the infection on to more vulnerable family members. There were limited prevention options in cramped homes and concerns regarding the course and long-term effects of the illness. As in other countries, adolescents were a lower priority for vaccination, compounding stress in some AGYW.

### 3.2. Community Insecurity during Lockdowns

#### 3.2.1. Community-Wide Insecurity and Gender-Based Violence

AGYW and KIIs described how gender-based violence and unsafe community conditions, already challenging pre-COVID-19, worsened over the course of the pandemic. With limited work and school access, young men in the community had little to occupy their time. AGYW narrated experiences of verbal harassment from young men in public spaces, which made parents wary of young women leaving the home. Community members described an uptick in alcohol and substance use among males, and an increase in petty thefts during lockdowns. Many young women described how their mobility had been curtailed after lockdowns due to safety concerns.

#### 3.2.2. Mistrust of the Government

Community insecurity was also described in terms of growing mistrust of government officials. Local police enforced stay-at-home orders, and sometimes resorted to physical violence to do so (described in one KII). Muslim community members, in particular, expressed distrust of national vaccination promotion efforts. Vaccination misinformation linked COVID-19 vaccines with population control efforts; rumors suggested that COVID-19 vaccinations contained contraceptive elements.

These qualitative data indicated that AGYW’s limited agency in terms of access to education, mobility, inequitable treatment in household responsibility and rules, and concerns about early marriage were linked with COVID-related psychological distress. Findings are confirmed by the quantitative data. The ‘agency during COVID-19 scale’ was significantly and positively associated with PHQ-8 scores (r = 0.31, *p* < 0.001) and GAD-7 scores (r = 0.27, *p* < 0.001), i.e., AGYW who experienced more restricted agency had higher levels of depression and anxiety, respectively. More restricted agency was also associated with greater frequency of *tenshun* (r = 0.34, *p* < 0.05) and *ghabrahat* (r = 0.32, *p* < 0.05).

### 3.3. New Opportunities and Supports Emerging during- and Post-COVID-19

#### 3.3.1. COVID-19 Lockdowns Promoted Family Time

Participants noted new opportunities for themselves, their families, and their communities that emerged during COVID-19 and its aftermath. For example, some AGYW felt that the COVID-19 stay-at-home orders brought family members closer and allowed them to spend more time with each other, talking and engaging in leisure activities.

#### 3.3.2. Financial and Material Resources

Although the initial COVID-19 lockdown in India was abrupt with limited planning or resources, participants felt that the governmental and community organizations provided more resources in subsequent phases of the pandemic. Governmental and NGOs provided funding and organized regular distributions of food, masks, and menstrual hygiene products. Participants noted that community lanes were cleaned by city officials more regularly.

#### 3.3.3. Employment and Leadership Opportunities

New employment opportunities for AGYW arose during COVID-19, as NGOs hired them to provide COVID-19 education, dispel misinformation concerning COVID-19 vaccinations, and assist community members in registering for the COVID-19 vaccine on the national electronic appointment platform. Other young women were enlisted to teach pre-school students lessons. AGYW received lesson links on their smart phone or tablet, and complemented these with materials from other sources. Payment for these activities helped young women at a time when families were financially struggling. It increased their confidence; many hoped to continue working in related areas post-COVID-19. The quantitative data support the positive association between AGYW’s work as COVID-19 educators or vaccine advocates and their mental health. Participating young women had lower GAD-7 scores (r = −0.18, *p* < 0.05).

## 4. Discussion

Our findings suggest that AGYW in low-income communities in Mumbai experienced compounding stressors during COVID-19 lockdowns, related to continuing their education, family financial instability, community safety, and overall reduced agency. Limited agency, in turn, was associated with AGYW depression and anxiety as well as local manifestations of psychological distress. These findings are consistent with the growing literature on worsening youth mental health during and after COVID-19, particularly for AGYW in marginalized communities [9,15,16,17,18,19,24,26,80,81,82].

Despite challenges, a subset of AGYW in our study leveraged new resources provided by government and NGOs to mitigate COVID-19 harm. Support of family members during lockdowns helped cope with stress [26,80,83,84]. New employment as a COVID-19 educator or vaccine advocate was associated with less anxiety for AGYW. Our findings support other studies documenting how participation in economic and peer support programming can mitigate hardships associated with health emergencies [21,22,85]. Approaches to develop and sustain resilience and agency are critical in the face of future pandemics and other health, climate, and humanitarian emergencies. Our findings were integrated into the planned peer support program, through an expanded module on mental health concerns and coping. We recommend that other community-based youth health and empowerment programs consider both the needs and assets that youth and their communities may have acquired over the course of the pandemic and incorporate these accordingly.

Strengths of our study include its mixed methods design that captures nuance concerning AGYW’s and adults’ experiences during COVID-19, as well as the opportunity to triangulate findings. The qualitative data enabled in-depth exploration of participants’ experiences, generated hypotheses for testing with the quantitative data, and offered key context for quantitative findings. The quantitative data allowed us to assess relationships between variables and quantify the extent of agency restrictions and mental health symptoms in the sample with widely used and validated tools (PHQ-8 and GAD-7). We also note limitations. The parent-study was designed before COVID-19 became a global emergency, although we were able to add COVID-19-specific questions to the needs assessment. Second, data collection for the needs assessment was cross-sectional, and towards the end of India’s COVID-19 emergency period (December 2021–July 2022). Given the potential for recall bias and the dynamic nature of COVID-19 in India, as in other countries, we cannot generalize our findings to other time periods or geographies. For example, the emergence of COVID-19 in India was marked by additional stressors related to the unknown nature of COVID, misinformation, and stigmatization of certain communities. [43]. As is the case with any cross-sectional design, our data are only reflective of a particular point in time. Finally, data were collected in urban, low-income communities in Mumbai, and cannot necessarily be generalized to experiences of young women in rural communities or other Indian states.

We found COVID-19-related constraints on agency were associated with higher levels of depression and anxiety for AGYW in low-income communities in Mumbai India. Participation in COVID-19 support/education activities was associated with reduced anxiety. Integrated and contextually relevant youth empowerment programs can address multiple dimensions (health, financial, social), and reduce depression and anxiety.

## Figures and Tables

**Table 1:** Survey domains and measures.

Domain	Measure
Demographic Information	- Age - Education - Religion - Birthplace (in or out of Mumbai) - Marital status and age at marriage - Mobile phone ownership - Household structure - Employment status - Food security [71]
Health concerns and treatment-seeking behaviors	- Sexual and reproductive, general health problems in the past 3 months [72,73] - Treatment sought for sexual and reproductive, general health problems in the past 3 months [72,73] - Receipt of sexual and reproductive health education [72] - Frequency of tenshun [74] - Frequency of ghabrahat [67,68] - GAD-7 [75] - PHQ-8 [76,77] - Sexual and reproductive health history [72]
Quality of and experiences with healthcare	- Type of healthcare accessed [72] - Reason for choosing healthcare provider [72] - Perceptions of healthcare provider/quality of healthcare [78] - Information received at healthcare provider’s office [72] - Sexual, reproductive, and mental health services requested [72]
Experiences during COVID-19	- Participation in COVID-19 community activities (attendance at COVID-19 education sessions, providing education to others on COVID-19 prevention, teaching younger students, helping distribute rations, helping others with online classes) [64,79] - Extent of COVID-19 restrictions on agency (burden of housework, ability to continue studies, time to meet with friends, activity restrictions, entertainment restrictions, employment, fear of early marriage, fear of community violence) [52,64,79]

**Table 2:** Key qualitative themes, sub-themes, and exemplary quotations.

Theme	Sub-Theme	Exemplary Quotations
Individual and Familial Stressors during COVID-19 Lockdowns	Difficulties continuing education and concerns about educational and employment aspirations	*…Online classes there are lots of problems like network problem, no silence, lots of disturbance. When you are at home and attending the class your family members too do not have that seriousness they ask you for water, tea during your class.* (22-year-old Unmarried Hindu AGYW) *The syllabus was changed when I was in 9th std, the 10th std syllabus was [taught] in the 9th std…Since then…we are …named called as virus generation children…And the government has declared that those who have passed their 12th std during the lockdown is not eligible for job…it adds to my tension.* (18-year-old Unmarried Muslim AGYW)
	Household food and financial scarcity contributing to distress and arguments	*My parents used to quarrel because of financial crisis, my mother used to worry a lot since we were 7 of us…Therefore, my father used to borrow money amounting 1000–2000 from his friends…[siblings and I] used to be scared…* (15-year-old Unmarried Muslim AGYW) *About adolescent girls, they are more affected mentally, because vaccine was not yet started for them. It creates more tension among adolescent and youth. You can say increased fear (ghabharaht), loss of weight and proper sleeplessness. Some said, loss of appetite, but I say, family don’t have enough money to buy food and vegetables and other day to day needs.* (Community Physician)
	Personal or familial experiences with COVID infection	*There is a cardiology hospital in [natal place] we had taken my dad…for angiography. When we were called again to the hospital there were 16 patients who were COVID positive…myself and my dad both were positive…It was 20 day isolation…I had breathing problem, at one point I thought I would not stay alive.* (21-year-old Unmarried Hindu AGYW)
Community-wide insecurity during COVID Lockdowns	Community-wide insecurity, concerns regarding gender-based violence	*I am scared that anything can happen because after 9 pm even when I go to throw garbage as a wife and a mother, even my life is at risk because there are few boys who sit there and I have seen and read news about rape that is happening every single day. Nowadays we are more scared of men than dogs…* (23-year-old Married Muslim AGYW)
	Mistrust of government officials	*Initially when lockdown had enforced…we were worried about income, but not much worried about COVID. But when 2 cases were found in our nearby [room], that time we all were having an enormous tension…Our home size is also very small, just 12X14 ft…everything was closed, and police was forcing us to stay at home. If someone goes outside, then they started to beat them.* (52-year-old Autorickshaw Driver and Father) *Awareness programs [are being run for] vaccination because many of them think that if they are vaccinated they will not conceive. Vaccination drive is run only to bring down the population rate, this is what people think…* (18-year-old Unmarried Hindu AGYW)
New Opportunities and Supports Emerging from COVID	COVID-19 lockdowns as a time for families to spend time together	*We as a family never had a chance to stay together but this was a lovely opportunity for us to be together…My mom never played ludo game but we as a family played this game together and had a good time…I learnt cooking. My papa also helped washing utensils. Everybody enjoyed.* (22-year-old Unmarried Hindu AGYW)
	Financial and material resources to improve conditions in the community	*Some NGOs and political parties provide the free sanitary pads to the adolescent girls. Asha health workers also visit the house regularly in the area to speak on vaccine…Government is providing free food grain to everyone for some time…* (Community Physician)
	New employment and leadership opportunities for young women	*I am teaching students in the community. I teach them online because during the pandemic schools were closed and so many students have missed their school…we receive links from school what is to be taught and accordingly we have to teach the students. We also have to make videos about whatever is taught…and I am also paid for it…* (20-year-old Unmarried Muslim AGYW)

**Table 3:** Characteristics of the adolescent girls and young women’s survey sample.

Variable	N (%)	Mean (Median; Range)
Education (highest standard/class completed)	Through 10th Standard: 104 (69.3)	9.54 years (10 years; 0–13)
Religion	Muslim 123 (82.0) Hindu: 22 (14.7) Other: 5 (3.3)	
Marital status	Unmarried: 122 (81.3) Married: 28 (18.7)	Age at marriage: 19.1 (19; 15–22)
PHQ-8 *	Minimal symptoms (0–4): 94 (62.7) Moderate to severe symptoms (5–24): 56 (37.3)	4.31 (3; 0–22)
GAD-7 *	Minimal symptoms (0–4): 88 (58.7) Mild to severe symptoms (5–21): 62 (41.3)	4.54 (4; 0–16)
Frequency of *tenshun*	Always/often: 26 (17.3) Sometimes/rarely: 92 (61.3) Never: 32 (21.3)	
Frequency of *ghabrahat*	Always/often: 13 (8.7) Sometimes/rarely: 82 (54.6) Never: 55 (36.7)	

* Higher scores on the PhQ-8 and GAD-7 indicate higher levels of depression and anxiety, respectively.

## Data Availability

The data reported in this manuscript come from the formative/non-trial phase of a clinical trial. The clinical trial is registered at clinicaltrials.gov under the number: NCT04307849. Deidentified data are available upon request to the corresponding author.

## Supplementary Materials

The following supporting information can be downloaded at: https://www.mdpi.com/article/10.3390/ijerph21081007/s1: qualitative interview guides and final qualitative codebook.
