# Supplementary material for: A Mixed Methods Exploration of Young Women’s Agency and Mental Health during COVID-19 in Low-Income Communities in Mumbai, India
Source: Int J Environ Res Public Health. 2024 Jul 31;21(8):1007. doi: 10.3390/ijerph21081007 (PMC11354834; doi:10.3390/ijerph21081007)
Supplement: Supplementary file 1 [file ijerph-21-01007-s001.zip › ijerph-3062345-supplementary.pdf]

Appendix

Adolescent girls and young women interview guide

I. Demographic questions
   a. Age
   b. Religion
   c. Education
   d. Occupation
   e. What neighborhood do you live in?
   f. Who do you live with at home?
   g. How long have you lived in Mumbai?

II. Health problems and treatment seeking
   a. Have you ever been sick or injured? Tell us about it.
   b. Tell us about the last time you were sick or felt unwell.
      i. What was wrong?
      ii. Did you go somewhere for treatment? If so, where did you go?
      iii. Why did you decide to go there?
      iv. How was your experience at that clinic/hospital/etc.?
      v. What worked well?
      vi. What did not work well?
      vii. What recommendations would you have for the doctor?

   c. Have you ever had any sexual or reproductive health problems?
      i. If yes, what was the problem?
      ii. Did you go somewhere for treatment? If so, where did you go? If not, why didn't you go for treatment?
      iii. If you went somewhere, why did you choose that clinic/hospital/etc.?
      iv. How was your experience there?

   d. Have you ever had any mental health issues (*tenshun*, *ghabrahat*)?
      i. If yes, what was the problem?
      ii. How did you feel? And how long did these feelings last?
      iii. What do you think caused these feelings?
      iv. Did you go anywhere for treatment? Why or why not?
      v. If you went for treatment, where did you go? Why did you choose that place?
      vi. What was your experience there?

III. Factors contributing to poor health
   a. What do you think causes poor health?
   b. What do you think causes poor sexual and reproductive health?
   c. What do you think causes poor mental health?

IV. Experiences during COVID-19
   a. How has life changed for you since the pandemic?
      i. Probe for schooling

        ii. Work (both in and out of the house)
        iii. Family (relationships and well-being)
        iv. Health (both sexual/reproductive/menstrual health and hygiene, and mental)
        v. Health-care seeking
        vi. Social support/socializing (including over phone/online)
    b. How have you been coping during the pandemic? Are there any activities that have helped you?
    c. Are there any programs that you have participated in to support you or your family during the pandemic? If so, please tell us about them.

V. Review intervention modules
    a. Probe for whether they think it would work or not, changes they would make
    b. Probe for COVID-specific considerations

Key informant interview guide

I. Background/demographic questions for people who work in the community:
    a. What is your role/title?
    b. What are your primary duties in this role?
    c. How long have you been working in this community?
    d. How long have you been working with adolescents/young adults?

II. Background/demographic questions for parents
    a. How old are you?
    b. What is your occupation?
    c. How long have you been living in the community?
    d. How many children do you have?
        i. How old are they?

III. Health status of adolescent girls and young women (for parents, probe around experiences specific to their children, what they have heard from others in the community)
    a. How is the health of AGYW in this community? Why do you say that?
        i. What are the main health problems you've observed or heard of?
    b. Where do AGYW go for healthcare?
        i. How would you rate the quality of care they receive?
        ii. What, if anything, could be done to improve the quality of care?
    c. What makes it harder for AGYW to get the healthcare they need?
    d. What makes it easier for AGYW to get the healthcare they need?
    e. How has COVID-19 impacted the health of adolescent girls and young women here?
        i. Probe for sexual, reproductive, and mental health
    f. Are there any programs that have supported the health needs of adolescents in this area during COVID? If so, please describe them.

IV. Social status of adolescent girls and young women
    a. What is your view about how young women grow up in the community?

        i. What are the community norms/rules for AGYW in this community?
b. Is the status of AGYW women changing in this community? Why or why not?
c. How has COVID-19 impacted the status of adolescent girls and young women?
    i. Probe for norms around mobility, relationships, timing of marriage, education, socializing, phone use
d. Are there any programs that have supported the social or education needs of adolescents in this area during COVID? If so, please describe them.

V. Review intervention modules
a. Health and social service provider training (the workshops)—how to approach adolescents/young adults and treat them fairly/not stigmatizing them, what the needs of adolescents/young adults are in the area, how to work together to improve the well-being of adolescents/young adults. Activities include discussion, team-building activities, sharing experiences, presentations from youth
b. Adolescent health club—groups of young women get together and learn about health and well-being, where to go to access resources/help, providing peer support to each other. Activities include: skits, interviewing others in the community, journaling, group discussions
    i. Probe for whether they think it would work or not, changes they would make
    ii. Probe for COVID-specific considerations
c. We are planning to have group discussions to present the intervention materials in more detail and see what needs to be changed. These discussions will probably be in late July/early August—are you willing to participate?
d. (For providers only) If you cannot participate in the group discussion sessions, would you like to participate in the service provider workshop (tentatively scheduled for November/December)?

Codebook for “Engaging youth, community members, and health care providers in implementing youth-centered care in Mumbai, India” Needs Assessment Phase

Draft version date: June 16, 2022

Code Name	Definition
**100. Community Context**	Descriptions of the community/state/national level and other people. Does not refer to personal experience of adolescent/young woman
100.1 Gender norms	Refers to the social and cultural context related to gender in/equity, societal/cultural norms of gender roles, power
100.2 Physical space/ environment	Description of physical space and the environment within the community
100.3 Covid	Description of what things were like in neighborhoods/chawl/community during Covid; excludes economics/livelihoods and use of prevention practices

100.4 Covid prevention practices	Use of masking or immunization to prevent Covid transmission or spread
100.5 Covid impact on economics and livelihoods	The impact of Covid on neighborhood/community economic status and livelihoods
100.6 Health care in the community	Availability of health care and health-seeking preferences of individuals in the community
100.7 Health issues in the community	Description of common health concerns that are prevalent in the community/neighborhoods; excludes sexual and reproductive health and mental health in the community
100.8 SRH in the community	Description of common sexual and reproductive health issues that people in the community have
100.9 Mental health	Description of mental health needs and concerns that exist in the community
100.10 Safety	Perceptions of safety or lack thereof in the community. Refers to crime, violence, arguments.
100.11 Sex work	Discussion of sex work in the study area/community, whether before Covid or as a result of Covid
100.12 Substance use and addiction	Discussion of substance use (alcohol, drugs, tobacco/paan, prescription drugs used in a way not intended) and addiction in the community
100.13 Use of mobile or other technology	Discussion of use of mobile technology by others (such a youth) in the community; attitudes or beliefs concerning the use of technology (helpful, causes problems, etc.)
100.14 Non-governmental community programs/ supports	Programs in the community provided by non-governmental organizations (NGO), or private citizens/foundations/politicians that provide material (food), health, health, social, or other support to individuals in the community
100.15 Governmental programs/supports	Government-funded programs (also referred to as “schemes”), strategies, or policies intended to support individuals in the community
100.16 Mistrust in the community	Description of mistrust in the community towards government, healthcare, or others.
100.17 Education	Description of access to education, norms related to education, educational/life aspirations.

**200. Individual experiences of adolescent girls and young women**	Personal experiences/beliefs of young women
200.1 Education	Young women's educational trajectories, feelings about school, reasons for continuing or leaving
200.2 Young women's work/livelihood	Paid work that young women are engaged in (could be within the home or outside the home), or previously engaged in
200.3 Aspirations for the future	Aspirations or plans for the future (employment, further study, marriage, etc.)
200.4 Gender norms, attitudes	Personal beliefs about gender roles--things that girls, boys, men, women do or are appropriate for them to do
200.5 Family context	Family structure, socioeconomic status and family members' livelihoods, and beliefs/norms
200.6 Family relationships	Descriptions of feelings about family relationships, support or lack thereof from the family
200.7 Social relationships (outside the family)	Description of relationship/interactions with peers, neighbors, individuals outside the household
200.8 SRH needs/concerns	Sexual and reproductive health issues or concerns, reproductive tract infections, STI/HIV risk/concerns, reproductive history (pregnancies, miscarriages, terminated pregnancies), sexual relationships/activities. Excludes menstrual health & hygiene
200.9 Menstrual health and hygiene concerns	Discussion of menstrual health and hygiene beliefs (whether they have restrictions during menstruation), health concerns related to menstruation, use of menstrual hygiene products or not
200.10 Non-SRH needs/concerns	Other health issues/concerns, such as seasonal colds, infectious disease, nutritional concerns
200.11 Mental health	Description of mental/emotional health and feelings (tenshun, anger, fear, ghabrahat or anxiety, etc.), self-harm, disordered eating patterns/body dysmorphia
200.12 Health & Support-seeking preferences	Where young women go for healthcare and/or social support, and why they go there
200.13 Health & Support-seeking experiences	Young women's experiences of accessing healthcare and social support and their rating of the quality of this care

200.14 Safety	Feelings about personal safety in the family or community; experiences of violence
200.15 Personal beliefs/experiences with Covid and Covid prevention	The individual young woman's experiences with Covid—whether she had Covid or anyone in her home had Covid, preventive practices that the young woman followed or not, whether the young woman is or plans to get vaccinated and why or why not. Whether or not family members living with the young woman are vaccinated and why/why not.
200.16 Use of mobile phone and other forms of technology	Discussion of whether and how the young woman uses mobile phones and other technology, including social media, and youtube, other website. Includes the use of technology for information, online schooling, connecting socially with others, learning new skills, etc.
**300. Pilot intervention**	Information/input on the planned pilot interventions
300.1 Content	Description of specific content or topics (patient communication, menstrual health, etc.) to be included in either the adolescent girls and young women's intervention or the healthcare provider/staff intervention
300.2 Activities	Description of activities (skits, discussions, interviews, etc) that should/shouldn't be part of either the young women's intervention of the healthcare provider/staff intervention, and whether or not they would work
300.3 Audience/participants	Recommendations for who should be included in the intervention
300.4 Implementation	Comments concerning the logistics of intervention implementation (timing, location, etc.)
300.5 Appropriateness/acceptability	Comments concerning the appropriateness and acceptability of intervention components/activities for the intended audience
**900. Overarching codes**	Should not be applied alone, but in combination with one or more of the codes above
900.1 Changes	Changes over time
900.2 Barriers/limitations	Factors that make something more difficult, or limit something

900.3 Facilitators/Resources to build on	Factors that make something easier, or are supportive
900.4 Recommendations	Participant recommendation
900.5 Great quote	A quote that is particularly illustrative or explains something particularly well
